# Investigation of the diurnal variation in bone resorption for optimal drug delivery and efficacy in osteoporosis with oral calcitonin

**DOI:** 10.1186/1472-6904-8-12

**Published:** 2008-12-04

**Authors:** MA Karsdal, I Byrjalsen, BJ Riis, C Christiansen

**Affiliations:** 1 Nordic Bioscience A/S, Herlev/, DK-2730 Herlev, Denmark; 2 CCBR, DK-2750, Ballerup, Denmark

## Abstract

**Background:**

Bone resorption displays marked diurnal variation. Reversible inhibition of bone resorption may result in best possible efficacy when bone resorption peaks. The aim of the study was to assess the pharmacokinetic (PK) and pharmacodynamic (PD) profiles of 0.8 mg of oral salmon calcitonin (sCT) and the effect of timing of drug intake.

**Methods:**

The study was a randomized, double-blind, double-dummy, placebo-controlled, phase I study to assess the pharmacokinetic (PK) and pharmacodynamic (PD) profiles of 0.8 mg of oral sCT in healthy postmenopausal women. Totally 81 subjects were included, aimed at investigation of a morning dose given at 8:00 (n = 42), a pre-dinner dose given at 17:00 (n = 20), and an evening dose given at 22:00 (n = 19). Plasma sCT concentrations and bone resorption (C-terminal-telopeptide of collagen type I (CTX-I)) was assessed.

**Results:**

Morning and pre-dinner dosing led to comparable concentration of sCT of 45 pg/ml, whereas there was a tendency towards lower Cmax for the evening dosing having a mean of 24 pg/ml. The maximum difference from placebo was observed 1 to 3 hours post-dose with a 40 to 50% suppression consequent to morning dose, and about 75% suppression after pre-dinner and evening dose, due to the increase bone resorption as a result of circadian variation.

**Conclusion:**

The study suggests that orally administered 0.8 mg of salmon calcitonin was effective in suppression of serum CTX irrespective of time of dosing. The pre-dinner dosing resulted in optimum efficacy response corresponding to an overall suppression of bone resorption by 25%.

**Trial registration:**

NCT00411125

## Background

Calcitonin (CT) is a 32 amino-acid hormone produced by parafollicular cells (C-cells) in the thyroid gland [1], secreted in response to excess calcium in the serum [2]. Binding of calcitonin to the calcitonin receptor on osteoclasts results in a reduction in a raid bone resorption [3-5]. Salmon calcitonin is among to most potent calcitonins, and belongs to the teleost/avian family[1]. Salmon calcitonin (sCT) is approved for the treatment of osteoporosis and other diseases involving accelerated bone turnover [6,7]. Preliminary evidence suggests that calcitonin may be a potential treatment for osteoarthritis that also entails increased osteoclast function [8-10].

Calcitonin treatment has until now been limited to either subcutaneous or intranasal calcitonin administration [6]. An increasing amount of studies have focused on providing an oral formulation, which includes but not are limited to these novel oral delivery methods [11-22]. 5-CNAC is the carrier responsible for absorption from the intestinal lumen into the bloodstream of calcitonin [6,8,9]. 5-CNAC is part of the Eligen technology, that employs low molecular weight compounds that interact weakly and non-covalently with proteins increasing its lipophilicity and thereby its ability to cross the gastrointestinal epithelium [23]. The carrier, 5-CNAC, enable systemic absorption of the drug via transcellular absorption [24-26]. This oral formulation in combination with sCT, has been demonstrated to be safe and efficaciously in a 3 month Phase II study in postmenopausal women [6].

Oral drug delivery of small peptides is hampered by an array of obstacles [12], including but not limited to degradation by proteolytic enzymes in the digestive tract and secondary intestinal uptake [12,13]. These difficulties are best illustrated by the complete lack of peptides in oral formulations approved by the FDA and EMEA. In addition to innovative strategies for improved uptake, [13] considering drug delivery based on knowledge of physiological bone turnover may further improve efficacy parameters. Diurnal variation is a well-established and important parameter of bone turnover, in which postprandial decreases in bone resorption result in an approximately 50% decrease in bone resorption compared to that of fasting individuals, and an equally large increase in bone resorption during the night [27,27-29]. This might attenuate the potential efficacy on bone resorption if co-administrated with food intake in the morning after the fasting period, in face of that of administration at a more optimal time of the day, i.e. in prior to bedtime or later in the evening where resorption peaks during the diurnal resorption period [27,27,28]. As bone resorption is decreased more than 50% after meal intake, additional benefits of anti-resorptives drugs may be limited, unless they provide sustained suppression in contrast to the completely reversible effect observed with calcitonin.

Bone resorption can be assessed by measurement of collagen type I degradation products. 90% of the protein of bone is collagen type I, that during bone resorption by osteoclasts is degraded by the cysteinse protease cathespin K[5]. This results in generation of CTX-I (C-terminal telo-peptide of collagen type I), that is a specific fragment of collagen type I exclusively generated by cathepisn K [30,31]. CTX-I fragments have been used as a surrogate measure of bone resorption for *in vitro*, preclinical and clinical studies [30,32].

The aim of the current study was to investigate the diurnal variation in bone resorption and to investigate the effect of oral calcitonin given as a morning dose, a pre-dinner dose, and an evening dose in healthy postmenopausal women. The absorption of calcitonin was assessed by measurement of plasma sCT concentrations, and bone resorption by the biochemical marker serum CTX-I (serum C-terminal telo-peptide of collagen type I). In addition, two different batches of oral calcitonin were investigated.

## Methods

### Drug substance

SMC021 A/C is an oral formulation of salmon calcitonin. The investigational drug consisted of 0.8 mg of recombinant sCT and 200 mg of 5-CNAC, {8-(N-2-hydroxy-5-chloro-benzoyl)-amino-caprylic acid)} a unimolecular enhancer of gastrointestinal peptide absorption developed by Emisphere Technology, Inc. and licensed to Novartis.

### Study design

The study was conducted in accordance with Helsinki Declaration II, and approved by local Ethical Committees (EudraCT number: 2006-002685-19), with the trial registration number NCT00411125. Written informed consent was obtained for all participants. The study was approved by the local Danish ethical committee, Copenhagen County.

The study was a randomized, double-blind, double-dummy, placebo-controlled study to assess the pharmacokinetic (PK) and pharmacodynamic (PD) profiles of two different variants of 0.8 mg of oral sCT (SMC021) and the effect of timing of drug intake in healthy postmenopausal women. The two different variants consisted of two production batches of the same product: Batch 002 was a pilot scale equipment batch and batch 010 a full scale production. The inclusion criteria requested that generally healthy ambulatory female volunteers were aged between 40–70 years and having passed a natural or surgical menopause at least 5 years before entering the study and being without diseases or medications known to affect bone metabolism.

The study was divided into 3 separate parts with timing of dosing and intake of meal at pre-defined time-points as outlined in Table 1. After consenting the women were allocated to participate in one of the parts.

**Table 1:** described the study design in the 3 parts with the time of meal and dosing of oral calcitonin.

Study Design: Timing of Dosing and Meal Intake												
	08:00	09:00	13:00	17:00	18:00	22:00	08:00	09:00	13:00	17:00	18:00	22:00

Part 1 Morning Dosing	Dose	Meal	Meal	-	Meal	-	End	x	x	x	x	x

Part 2 Pre-dinner Dosing	x	x	Meal	Dose	Meal	-	-	Meal	Meal	End	x	x

Part 3 Evening Dosing	x	x	x	x	Meal	Dose	-	Meal	Meal	-	Meal	End

(-) Denotes study period with no action. (X) Denotes not part of study period.

Part 1 included a total of 42 subjects and was aimed at investigation of PK and PD of two variants of 0.8 mg of sCT and placebo given in the morning at 8:00. At the start of the study, subjects were randomized to receive active treatment (the two production variants) and placebo in a specific order. Part 1 had 3 treatment periods of 3 days, with study drug intake in the morning of each day. The wash-out period was at least 3 days between treatment periods. After an overnight fast, subjects received the dose of drug with 200 ml of water and was sampled immediately before drug intake, and at 5, 10, 15, 30, 45 minutes, 1, 1 1/2, 2, 2 1/2, 3 hours, and every hour until the second dosing 24 hours after first dose. Meals were served at scheduled time points as outlined in Table 1. No other food consumption at any other time point was allowed, but intake of water was allowed from 1 hour after dosing.

Part 2 included a total of 20 subjects and was aimed at investigation of PK and PD of an oral dose of 0.8 mg of sCT (batch 010) and placebo given pre-dinner at 17:00. At the start of the study, subjects were randomized to receive active treatment and placebo in a specific order. The study included 2 treatment periods with a single dosing each and with a wash-out period of at least 3 days between treatment periods. On the first day of treatment, the subjects arrived at the clinic during the morning, had a meal at 13:00 and received the first dose at 17:00. Additional meals were served at the scheduled time points, and the 24-hour blood sampling schedule was similar to that described in Part 1.

Part 3 included a total of 19 subjects and was aimed at investigation of PK and PD of an oral dose of 0.8 mg of sCT (batch 010) and placebo given in the evening at 22:00. The randomization, treatment periods, and wash-out period were similar to Part 2. On the first day of treatment, the subjects arrived at the clinic at dinner time, had a meal at 18:00 and received the first dose at 22:00. Additional meals were served at the scheduled time points, and the 24-hour blood sampling schedule was similar to that described in Part 1.

The plasma and serum samples were stored at -20°C until analysis. For PK assessment, plasma sCT was measured in the blood samples collected in the period of 0–4 hours after drug intake.

The concentration of plasma sCT was measured by a chemiluminescence-based immunoassay as previously described [6]. Values measured below the lower limit of quantification of 2.5 pg/ml was assigned the value of 2.5 pg/ml. The assay was a two-site immunometric type employing two antibodies, one biotinylated and the other acridium labeled. Specificity has been tested against synthetic fragments of sCT and against human as well as eel calcitonin and negligible interaction has been found over the range of standard curve. The lower limit of quantification (LLOQ) was 2.5 pg/ml. The quality control samples, ranging from 2.5 pg/ml to 700 pg/ml, were prepared daily and measured in 3 to 5 replicates. The overall accuracy and precision (CV) of the control samples measured on 11 different days was 101.3% and 10.1% for 2.5 pg/ml concentration and 94.3% and 6.0% for 700 pg/ml concentration, respectively.

The Serum CTX-I test is a sandwich enzyme enzyme-linked immunosorbent assay (ELISA) employing two monoclonal antibodies both recognizing the C-telopeptide of the α1-chain in type I collagen [33]. The monoclonal antibodies, i.e. MAb F1103 and MAb F12, recognize the eight amino acid sequence EKAHD-β-GGR, where D- β -G denotes an isomerised bond between aspartate and glycine, and both antibodies require the presence of a free C-terminal arginine for binding. Cathepsin K, secreted by the osteoclast, is responsible for the proteolytic cleavage exposing the free C-terminal arginine [30]. The sandwich construction assures that only cross-linked di-peptides, i.e. EKAHD-β-GGR × EKAHD-β-GGR, are measured by the Serum CTX-I ELISA. The measuring range is 0.020–3.380 ng/ml, and in this range the intra- and interassay coefficient of variation is < 3.0 and < 10.9%, respectively, and the dilution recovery 103%. The reference range (mean (95% confidence interval) for postmenopausal and premenopausal women as well as men is 0.439 ng/ml (0.142 – 1.351 ng/ml), 0.287 ng/ml (0.112 – 0.738 ng/ml), and 0.294 ng/ml (0.115 – 0.748 ng/ml), respectively, according to the manufacturer (Immunodiagnostic Systems Nordic, Herlev, Denmark)[33].

### Statistical analysis

The sample size was calculated separately for the three parts of the study. With the primary objective of investigating the PK profiles of the two variants of sCT in Part 1, a sample size of 42 subjects was necessary to have at least 85% power to reject the null hypothesis that the absolute difference in the log_e_-transformed C_max _of the two variants was above 0.693 using a one-sided t-tests at the 5% significance level, based on a two-arm cross-over design. Transformed back to the original scale, the null hypothesis corresponds to ratio less than 0.5 or greater than 2.0. The alternative hypothesis was that the difference in mean loge-transformed C_max _between the two variants was 0.05 (that is, the C_max _of the batch 010 variant was 95% of the batch 002 variant and that the between-subjects standard deviation on the log_e _scale was 1.0. Similar sample size calculations applied for AUC_0–4 hrs_.

For Parts 2 and 3, a sample size of 20 subjects was necessary to have at least 90% power to reject the null hypothesis that the difference in the serum CTX AUC_0–24 hrs _between sCT and placebo was less than 0.150 using a one-sided t-test at the 5% significance level using a two-arm cross-over design. The between-subjects common standard deviation was assumed to be 0.11.

No randomization of subjects was performed to Parts 1, 2 or 3 of the study, therefore comparisons between the three parts should be made with care as they may be affected by bias.

The trapezoidal method was applied for the calculation of AUC_0–4 hrs _of plasma sCT and relative change in serum CTX after dosing. The relative value of serum CTX was calculated as percentage of the individual pre-dose value within each treatment period. As a post-hoc unplanned analysis, the relative change of serum CTX was determined as 100% minus the relative value of serum CTX. The AUC of plasma sCT, and the time course data of plasma sCT, serum CTX, and relative value of serum CTX were logarithmically transformed to obtain normality and symmetry of variances.

Comparison of the two variants of sCT on the pharmacokinetic parameters of sCT C_max _and sCT AUC_0–4 hrs _investigated in part 1 was performed in a linear mixed effect model with parameter as response variable and variant (var002, var010) and treatment sequence (1,2,3) as fixed effects and subject as random effect. In each dose time group the treatment response on sCTX AUC_0–24 hrs _was assessed in a linear effect model with treatment group (var002, var010, placebo) and treatment sequence (1,2,3) as fixed effects and subject as random effect. Comparison of the parameters of sCT C_max _and sCT AUC_0–4 hrs _in the active treatment groups among the dose time groups was performed in a linear effect model with parameter as response variable and dosing time (part 1,2,3) and sequence (1,2,3) as fixed effects. In the comparison of the active treatment response of var010 on sCTX AUC_0–24 hrs _among the dose time groups, first the placebo-corrected values were calculated by subtracting the placebo AUC from the treatment AUC for each individual subject. The placebo-corrected AUC was then compared among the dose time groups in a linear effect model having dosing time (part 1,2,3) as fixed effect. The significance levels were Tukey-Kramer adjusted in the multiple comparisons.

A difference was considered significant if p-value was less than 5%. All statistical calculations were performed using the SAS software package (release 9.1, SAS Institute Inc., Cary, NC, USA).

## Results

The age characteristics of the study participants are given in Table 2. The subjects were aged between 57 and 71 years, and comparable between the three parts of the study.

**Table 2:** Age characteristics of study population

	Part 1 *n *= 42 Morning Dosing	Part 2 *n *= 20 Pre-Dinner Dosing	Part 3 *n *= 19 Evening Dosing
Age (years)	65.5 (3.8)	64.6 (4.2)	64.4 (2.9)

Values are mean (SD)

**Figure 1:**
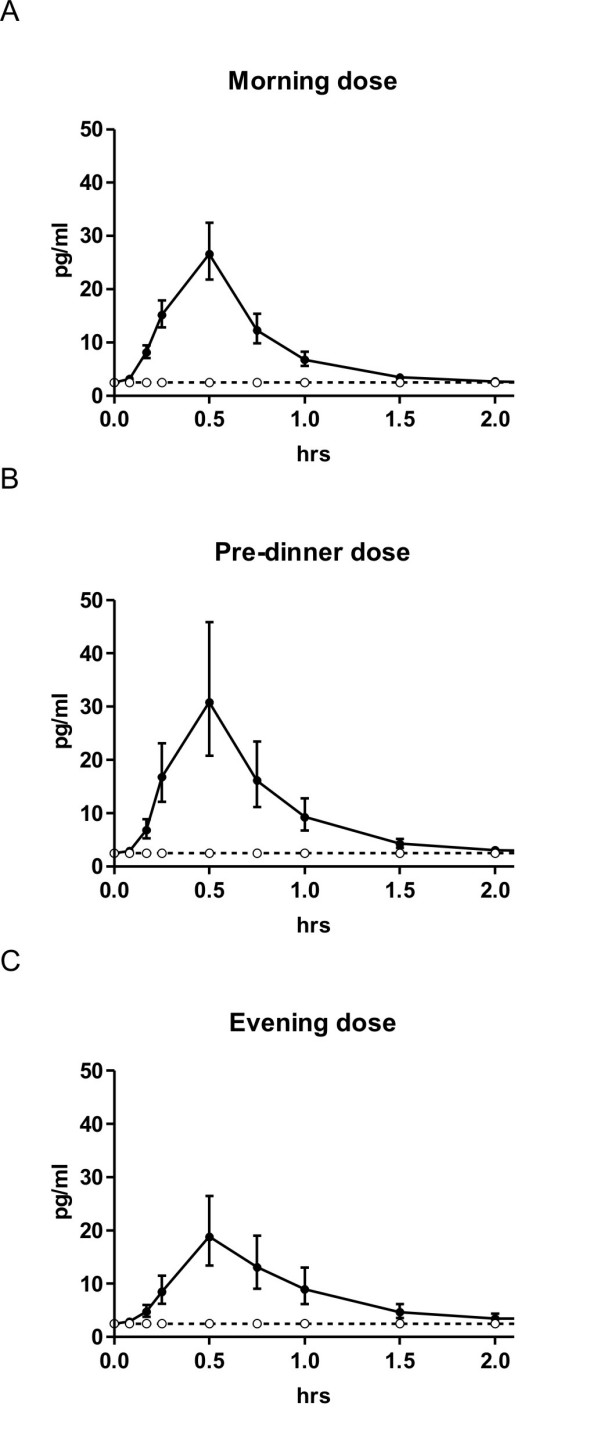
**Time course of plasma sCT in each time dose group.** Full line shows the results of 0.8 mg of oral sCT and the dotted line that of placebo. Morning dose (*n *= 42); pre-dinner dose (*n *= 20); and evening dose (*n *= 19). Values given are geometric mean ± 1 SEM.

**Figure 2:**
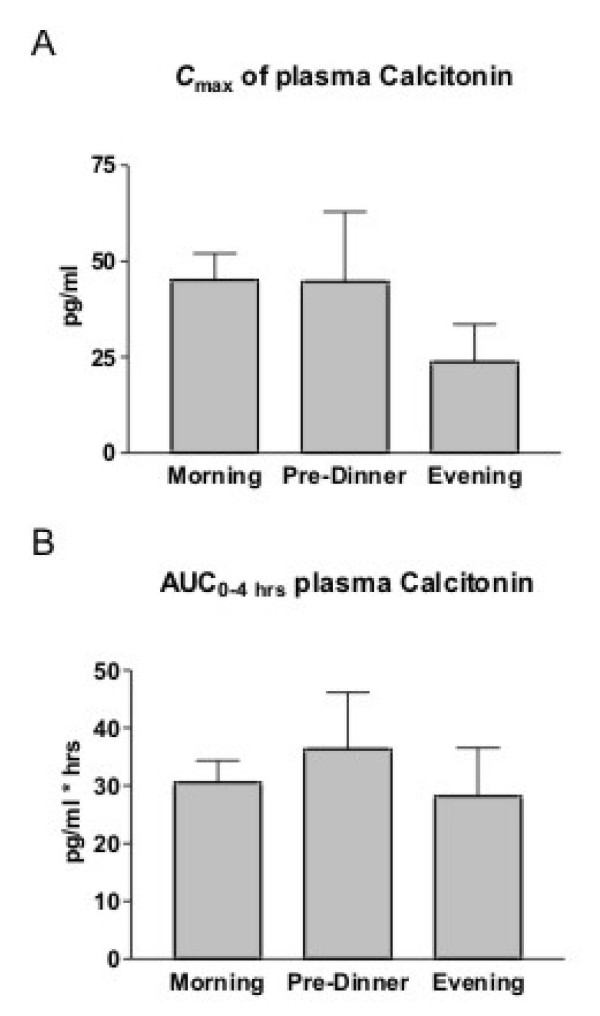
**C_max _and AUC_0–4 hrs _of plasma sCT in the 4 hours following one single dose of 0.8 mg of oral sCT in each time dose group corrected for AUC of the placebo groups.** Morning dose (*n *= 42); pre-dinner dose (*n *= 20); and evening dose (*n *= 19). Values given are geometric mean ± 1 SEM.

**Figure 3:**
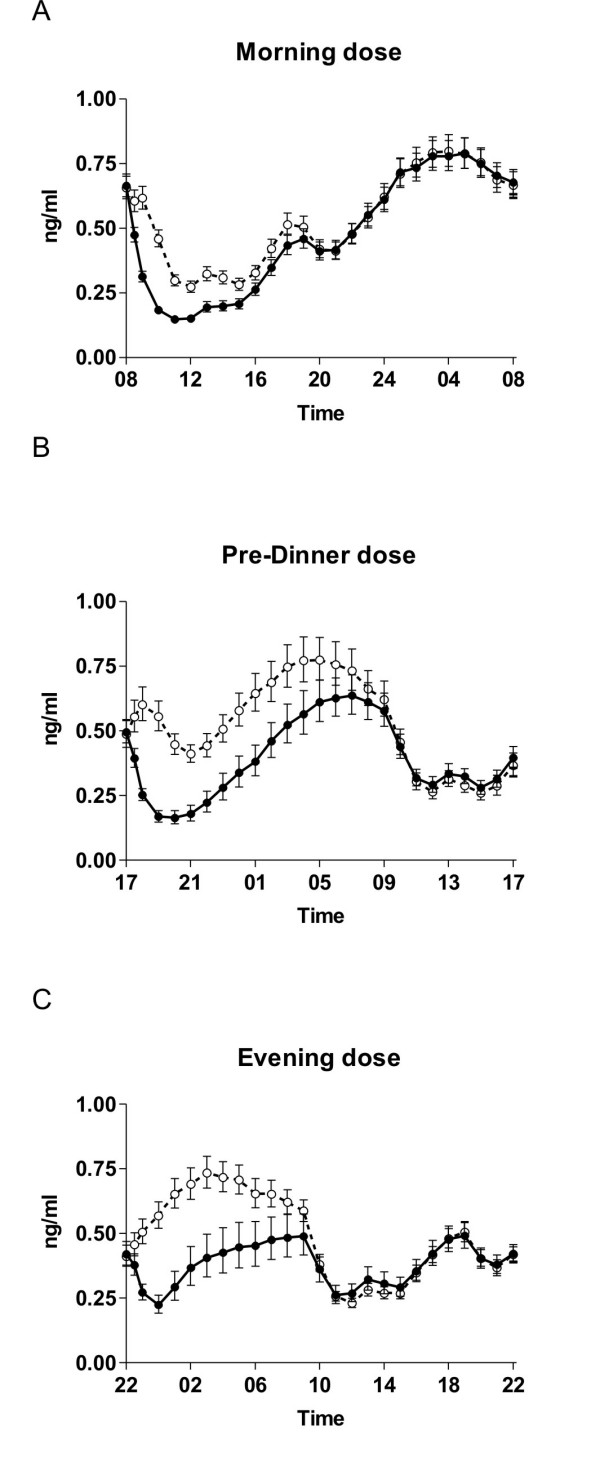
**Time course of absolute levels of serum CTX in each time dose group.** Closed circles show the results of 0.8 mg of oral sCT and the open circles that of placebo. The figures show results of the full follow-up period of 24 hours. Morning dose (*n *= 42); pre-dinner dose (*n *= 20); and evening dose (*n *= 19). Values given are geometric mean ± 1 SEM.

The effect of time of dosing on the absorption of SMC021 is shown in Figure 1. The sCT appeared rapidly in plasma with a median time to reach maximum (T_max_) at about 30 minutes post-dose irrespective of dosing time (Table 3). Following T_max _the plasma concentration returned to baseline approximately 2 hours after dosing. Morning and pre-dinner dosing gave a maximum geometric mean C_max _of 45 pg/ml, whereas there was a tendency towards a lower C_max _of 24 pg/ml for the evening dosing although this was statistically non-significant (ANOVA p = 0.14; morning versus evening dosing p = 0.05). The total exposure, AUC_0–4 hrs _of plasma sCT in the period between dosing and 4 hours post-dose was in the range of 28 to 36 (pg/ml × hrs) and was not influenced by time of dosing (Figure 2, Table 3) (p = 0.61). The two variants were found to be similar in terms of the pharmacokinetic parameters of AUC_0–4 hrs _and C_max _based on the pre-specified requirements in the protocol: the 95% confidence interval for the ratio in log-transformed AUC_0–4 hrs _and C_max _between the two variants was 82% to 125%, and 69% to 120%, respectively, both within the 50% to 200% defined in the protocol. There was no statistically significant effect of treatment sequence and also the pharmacodynamic effect of sCTX AUC_0–24 hrs _was comparable between the 2 variants (p = 1.00). The efficacy of the administered SMC021 was assessed by the pharmacodynamic profile of bone resorption measured by the biochemical marker of CTX in serum samples obtained until 24 hours after dosing. Overall, dosing with SMC021 resulted in a significant suppression of serum CTX over placebo irrespective of dosing time (Figure 3). Following the morning dose of SMC021, serum CTX decreased to a nadir 3 to 4 hours post-dose (Figure 3). At the time of maximum suppression, the level of serum CTX was decreased to 22% relative to the pre-dose value. The maximum difference of 40 to 50% from placebo was found 1 to 2 hours after dosing. A return to the placebo level was seen 12 hours post-dose following morning dosing. A higher suppression of serum CTX was found for pre-dinner and evening dosing with a maximum difference of about 75% from placebo at 1 to 3 hours after dosing due to the circadian variation in serum CTX observed in the placebo group. In addition the pre-dinner dose tended to have a prolonged period of action with a remission to the placebo level seen 16 hours post-dose. There were no statistical differences between the two variants on pharmacodynamic parameters.

**Table 3:** Pharmacokinetic profile

	Part 1 *n *= 42 Morning dosing	Part 2 *n *= 20 Pre-dinner dosing	Part 3 *n *= 19 Evening dosing
C_max _(pg/ml)	45.1 (39.1–52.0)	44.7 (31.8–62.9)	23.8 (16.9–33.5)

T_max _(minutes)	30 (15–30)	30 (15–30)	30 (30–33.8)

AUC_0–4 hrs_	30.6 (27.2–34.4)	36.3 (28.6–46.1)	28.1 (21.6–36.6)

AUC_0–4 hrs _and C_max _is given as geometric mean (mean ± 1SEM range) and T_max _as median (25–75 percentile)

**Figure 4:**
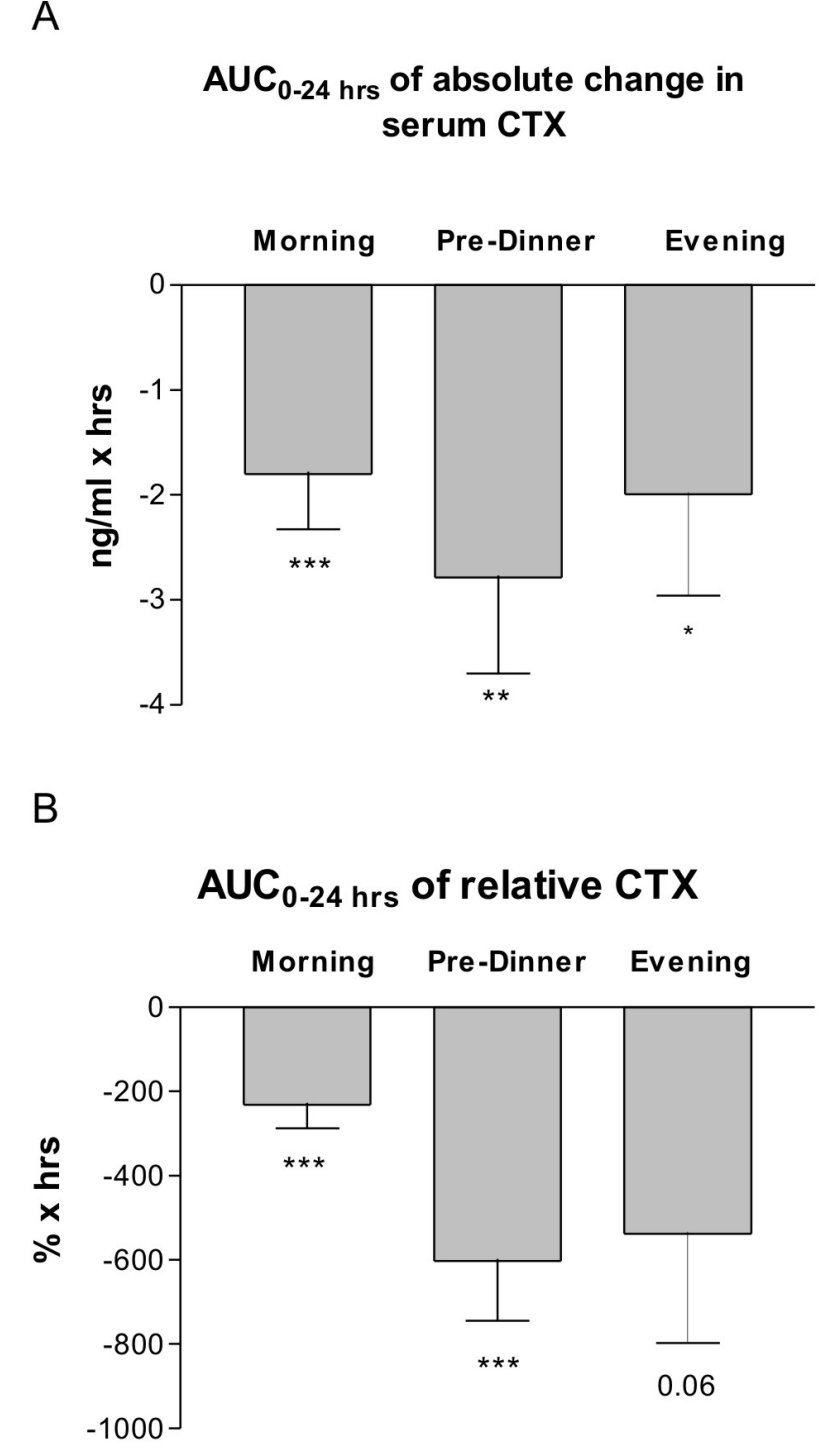
**AUC of absolute (A) and relative (B) change in serum CTX in the 24 hours following one single dose of 0.8 mg of oral sCT in each time dose group corrected for placebo AUC of the individual subject.** Morning dose (*n *= 42); pre-dinner dose (*n *= 20); and evening dose (*n *= 19). Values given are geometric mean ± 1 SEM. The level of significance denotes difference from placebo: **p < 0.01; *** *p *< 0.001.

The total response of dosing with SMC021 was assessed as AUC of change from the baseline levels during the 24-hour follow-up period. The placebo-corrected AUC_0–24 hrs _related to the morning dose of SMC021 was statistically highly significant (p < 0.001) with a mean value of -232 (% × hrs) (Figure 4). This decrease would correspond to a permanent overall suppression of bone resorption by 10%. The pre-dinner dose was twice as effective in suppression of serum CTX giving a mean AUC_0–24 hrs _of -603 (% × hours) (p < 0.001) corresponding to an overall suppression of 25%. The AUC_0–24 hrs _of the evening dose was at the same level of magnitude at -538 (% × hrs) (p = 0.06) although there seemed to be a higher inter-individual variation in the response.

## Discussion and conclusion

We have investigated the effect of drug timing on the bioavailability and pharmacokinetic profile of a single dose of 0.8 mg of salmon calcitonin given orally with the aim to determine the best timing of dose for optimal bone marker efficacy. We asked the question if dosing in a not fully fasting state, i.e. pre-dinner, or before bedtime as compared to an overnight fasting state before breakfast, would result in clinical efficacy.

In this closely monitored study, the results of the placebo groups clearly show the intrinsic diurnal variation in bone resorption with suppression consequent to meal intake. A decrease in bone resorption could be observed 1 hour postprandial both after the breakfast meal at 09:00, lunch at 13:00, and dinner at 18:00. These observations are in line with those previous observed post prandially [27,28,34]. Importantly, the postprandial decrease in bone resorption was largest in the morning, suggesting that longer fasting periods are needed for obtaining the full effect on bone resorption post prandially.

The absolute levels of bone resorption were different at the different time of day, in accordance with the diurnal variation [29]. Calcitonin C_max _or AUC_0–4 hrs _was similar following dosing in the morning and evening, although there was a tendency towards lower absorption in the evening. This did however not reach statistical significance, maybe in part due to the inter-individual variation. In contrast, this resulted in higher decreases in bone resorption taken when bone resorption was higher, i.e. before dinner and during the night. This may partly be due to lack of the postprandial decrease in bone resorption after the long fating period in the morning and partly because of inhibition of bone resorption at higher levels, i.e. at peak intensities during the night, compared to that of the morning.

With respect to safety issues, this current oral formulation of sCT have previously been evaluated [6] in a three months safety and efficacy study. There were no safety concerns with this oral formulation.

With respect to longer term efficacy, several parameters need to be evaluated. Bone formation in healthy individuals is tightly coupled to bone resorption, in which an equal amount of bone formation is made in response to that of bone resorption [35,36]. Normally, a secondary decrease in bone resorption is seen with anti-resorptive drugs secondary to that in bone resorption [30,37]. Interestingly, with this present oral formulation an inhibition in bone resorption without the secondary effect on bone formation was observed, in the 3 months efficacy study [6]. This might be as a consequence to the transient inhibition of bone resorption in contrast to other treatments with sustained inhibition [36], although this remains speculative. Thereby, addition dosing may not result in the theoretical additive effect, as a consequence to a secondary inhibition of bone formation.

In the current study, bone resorption was evaluated by CTX-I, a marker of bone resorption [38]. An extensive amount of research have been conducted with CTX-I, in which effects on food intake and circadian variation are well established parameters [27,29,34,39], and changes in CTX-I accurately reflects changes in bone resorption both in vitro and in vivo [35,40]. Changes in bone resorption markers, in response to food intake have been reported for other markers of bone resorption, whereas markers of osteoclast number such as TRAP does not change [41]. Taken together, even though that we in the present study have used the most well-established bone resorption markers, other markers of osteoclast number and function such as TRAP, NTX and cathepsin K [30] may in addition shed addition light into the mode of action of sCT on bone resorption.

For optimization of the efficacy of anti-resorptive drugs, with special emphasis on completely reversible interventions such as calcitonin, the timing of drug dosage is important. The diurnal variation in bone resorption, which is tightly associated with meal intake, is principal in bone biology, and allows for timing of dosage at peak bone resorption periods. Our data suggest that pre-dinner dosing was more effective in achieving absorption (in fasting conditions) and in the suppression of serum CTX in comparison with morning or bed-time dosing. These investigations and data are instrumental in understanding and utilizing natural circadian rhythms for optimising drug efficacy.

In addition to the primary objectives we investigated the comparability of two production batches of oral calcitonin in terms of absorption and surrogate efficacy markers. The two variants investigated were shown to be comparable in terms of absorption and of effect on bone resorption, serum CTX.

In conclusion, our data suggest that sCT given pre-dinner results in improved absorption and changes in serum CTX compared to that of dosing in the morning or at bed-time. Further clinical studies are needed to investigate these promising data for the treatment of osteoporosis.

## Competing interests

All authors are full time employees of Nordic Bioscience, a company engaged in the development of biochemical markers of bone and cartilage turnover and the development of oral calcitonin. MAK and CC hold stocks in Nordic Bioscience.

## Authors' contributions

CC and BJR designed the study and reviewed the last version of the manuscript. IB performed statistical analysis and participated in drafting of the manuscript. MAK analyzed the data, drafted the first manuscript and finalized the last version of the manuscript. All authors read and approved the final manuscript.

## Pre-publication history

The pre-publication history for this paper can be accessed here:


